# Adult mice transplanted with embryonic retinal progenitor cells: New approach for repairing damaged optic nerves

**Published:** 2012-11-12

**Authors:** Jang-Hyeon Cho, Chai-An Mao, William H. Klein

**Affiliations:** Department of Biochemistry and Molecular Biology, The University of Texas MD Anderson Cancer Center, Houston, TX

## Abstract

**Purpose:**

Retinal ganglion cell (RGC) death and optic nerve degeneration are complex processes whose underlying molecular mechanisms are only vaguely understood. Treatments commonly used for optic nerve degeneration have little long-term value and only prolong degeneration. Recent advances in stem cell replacement therapy offer new ways to overcome RGC loss by transferring healthy cells into eyes of afflicted individuals. However, studies on stem cell replacement for optic nerve degeneration are hampered by limitations of the available animal models, especially genetic models. We have developed a mouse model in which RGCs are genetically ablated in adult mice with subsequent degeneration of the optic nerve. In the study reported here, we used this model to determine whether embryonic retinal progenitor cells (RPCs) removed from donor retinas when RPCs are committing to an RGC fate could restore lost RGCs.

**Methods:**

We used the RGC-depleted model as a host for transplanting donor green fluorescent protein (GFP)–labeled RPCs from embryonic retinas that are maximally expressing *Atoh7*, a basic helix–loop–helix gene essential for RGC specification. Dissociated GFP-labeled RPCs were characterized in situ by immunolabeling with antibodies against proteins known to be expressed in RPCs at embryonic day (E)14.5. Dissociated retinal cells were injected into the vitreous of one eye of RGC-depleted mice at two to six months of age. The injected and non-injected retinas were analyzed for gene expression using immunolabeling, and the morphology of optic nerves was assessed visually and with histological staining at different times up to four months after injection.

**Results:**

We demonstrate the successful transfer of embryonic GFP-labeled RPCs into the eyes of RGC-depleted mice. Many transplanted RPCs invaded the ganglion cell layer, but the efficiency of the invasion was low. GFP-labeled cells within the ganglion cell layer expressed genes associated with early and late stages of RGC differentiation, including *Pou4f1*, *Pou4f2*, *NFL*, *Map2*, and *syntaxin*. Several GFP-labeled cells were detected within the injected optic nerves of RGC-depleted mice, and in most cases, we observed a significant increase in the thickness of the RPC-injected optic nerves compared with non-injected controls. We also observed more bundled axons emanating from RPC-injected retinas compared with RGC-depleted controls.

**Conclusions:**

The results offer a new approach for regenerating damaged optic nerves and indicate that a significant number of E14.5 RPCs are able to differentiate into RGCs in the foreign environment of the adult retina. However, the proportion of RPCs that populated the ganglion cell layer and contributed to the optic nerve was not sufficient to account for the increased thickness and higher number of axons. The results support the hypothesis that the injected E14.5 RPCs are contributing autonomously and non-autonomously to restoring damaged optic nerves.

## Introduction

A World Health Organization 2001 report stated that between 40 and 45 million people worldwide are blind. A significant portion of those have some form of optic nerve degeneration [1,2]. Although the problem is immense, therapeutic approaches to regenerating damaged optic nerves lag substantially behind those used for other types of ocular disease. In addition, animal models currently available for developing new therapeutic strategies suffer from several serious limitations [1].

We recently developed a robust genetically engineered adult mouse model of retinal ganglion cell (RGC) loss and optic nerve degeneration based on genetic ablation. We took advantage of the *Pou4f2* gene, which is essential to RGC differentiation and is expressed in RGCs throughout life. Adult mice were generated whose genomes harbored a conditional *Pou4f2* allele containing a *floxed-lacZ-stop-dta* cassette and a *CAGG-Cre-ER* transgene (Dta mice). When mice at different ages were administered tamoxifen by intraperitoneal injection, the result was rapid RGC loss, reactive gliosis, progressive degradation of the optic nerve over a period of several months, and visual impairment [3]. Although the efficiency of the toxin-mediated cell death was high and completely ablated all *Pou4f2*-expressing RGC, the retinas maintained their structural integrity even in two-year-old mice [3]. The Dta mouse model is particularly relevant for RGC death and optic nerve degeneration in human retinal pathologies, where the degeneration of non-RGC types does not typically occur until advanced stages of pathogenesis. The events that we observed in Dta mice are hallmarks of progressive optic nerve degeneration observed in human retinal degenerative diseases and demonstrate the validity of this model for use in developing treatment strategies for optic nerve degeneration.

A promising new approach for optic nerve degeneration involves stem cell replacement strategies [1,2]. Unfortunately, adult retinal stem cells are not yet available to use for cell transplantation. Indeed, whether retinal stem cells even exist in the adult retina is an open question. In contrast, embryonic retinas offer a readily available source of progenitor cells for all retinal cell types. Herein, we focus on the progenitors of RGCs.

In the mouse, as in other vertebrates, RGCs are the first cells to differentiate from retinal progenitor cells (RPCs) [4]. For RGC commitment to occur, a population of *Pax6*-expressing RPCs must downregulate the Notch signaling pathway, exit the cell cycle, and express the proneural bHLH gene *Atoh7 (Math5)* [5]. These events render *Pax6/Atoh7*-expressing RPCs competent for specifying the RGC lineage. Although *Atoh7* null retinas lack only RGCs, lineage analysis has shown that in addition to RGCs, *Atoh7*-expressing RPCs can give rise to non-RGC cell types [6,7]. In early stages of development, the majority of *Atoh7*-expressing RPCs commit to an RGC fate, but other cell types are represented in the *Atoh7*-expressing cell lineage. At later stages, however, many *Atoh7*-expressing RPCs commit to non-RGC types of cells, suggesting that *Atoh7* has functions in forming other retinal cell types. In the mouse retina, the earliest signs of overt RGC differentiation are the downregulation of *Atoh7* expression and the onset of expression of two key transcription factors essential for normal RGC differentiation, the Pou domain factor Pou4f2 (Brn3b) and the Lim domain factor Isl1 [8,9]. Pou4f2, Isl1, and most likely other early-expressing transcriptional regulators activate a hierarchical RGC gene regulatory network consisting of additional downstream transcription factors and signaling molecules that feed back to RPCs to maintain the correct balance of proliferating RPCs and differentiating RGCs [8].

Although attempts using various stem cell or progenitor cell populations have yet to produce effective methods for restoring RGCs [10,11], work on repairing photoreceptor cells has led to impressive advances. Although mouse models of RGCs are limited, several genetic mouse models are available in which to study the transfer of photoreceptor progenitor cells [12]. These mice have specific mutations in various genes that result in rod or cone photoreceptor cell death. Moreover, whereas RGCs are projection neurons whose axons must extend long distances, photoreceptor cells make short axonal connections. Replacing defective photoreceptor cells in *rho* mutant mice leads to improved light-mediated behavior [13]. MacLaren et al. [14] reported that rod photoreceptor cells were restored in *rd3* mice by transplanting photoreceptor precursors. The transplanted progenitors differentiated into rod photoreceptors, generated photosensitive rod segments, and made proper connections to bipolar cells, which resulted in functional synapses. The repaired retinas led to improved visual function. A key element for the success of this approach was choosing the developmental time when the number of rod progenitors was the highest. This is when rod progenitors express *Nrl*, a leucine zipper factor required for rod development [15]. This allowed green fluorescent protein (GFP)–labeled *Nrl*-expressing cells to be purified and used for the transplantation experiments.

We used MacLaren et al.’s [14] paradigm in our study. By isolating RPCs at E14.5, when the numbers are maximal, and transferring the progenitor cells into the eyes of Dta mice, we demonstrate that GFP-expressing RPCs differentiated into RGCs and contributed to repairing damaged optic nerves. Although RPC transplantation led to only small numbers of differentiated RGCs, the approach offers new possibilities for pursuing stem cell therapy for optic nerve degeneration.

## Methods

### Animals

All animal procedures and handling followed the USA Public Health Service Policy on Humane Care and Use of Laboratory Animals and were approved by the Institutional Animal Care and Use Committee at the University of Texas MD Anderson Cancer Center.

### Host strains

*Pou4f2^lacZ-dta^* heterozygous [16] and *CAGGCre-ER^TM^* heterozygous [17] mice were bred with each other, and pups from the mating pairs were used as host mice for the transplant procedures. Genotypes were confirmed with polymerase chain reaction. Tamoxifen (Sigma, St. Louis, MO) was administered to one-month-old mice by intraperitoneal injection once a day for five consecutive days at a concentration of 5 mg/40 g of bodyweight in corn oil (Sigma). One to five months after the last tamoxifen injection, the host animals received an injection of dissociated RPCs in the right eye (see the next section). Mice were euthanized two to 16 weeks after the dissociated RPCs were transplanted.

### Donor cell preparation and transplant procedures

Homozygous GFP mice (EGFP/EGFP) [18] were bred with homozygous *Atoh7-HA* mice [19] to obtain double heterozygous offspring. Embryonic retinas were harvested at embryonic day (E) 14.5 from the mating pairs, and at least ten retinas were combined and dissociated with the Worthington Papain Dissociation System (Worthington Biochemical Co, Lakewood, NJ). Complete dissociation of the retina was confirmed with fluorescence microscopy, and the RPCs were counted before the transplant procedure. Trypan blue (Sigma) was used to determine the number of dead cells after papain dissociation. Cell transplantation was performed under an operating microscope equipped with an intraocular injection kit (World Precision Instruments, Sarasota, FL). Briefly, mice were anesthetized with an intraperitoneal injection of 0.5 ml of 2,2,2,-tribromoethanol (20 mg/ml). A 30-gauge needle (BD Bioscience, Franklin Lake, NJ) was used to make a hole on the sclera, and then the tip of a 1.5-cm 34-gauge needle was inserted through the sclera. Two μl of the donor cell suspension (up to 1×10^5^ cells) was slowly released into the intravitreal space of the eye. A phosphate-buffered saline (PBS) buffer-injected eye was used as the injection control.

### Immunohistochemical analysis

Dissociated RPCs from the donor mice were placed on poly-lysine-coated adhesion slides (Fisher Scientific, Waltham, MA) and fixed in 10% neutral buffered formalin (10% NBF) for 1 h. Endogenous peroxidase activity and nonspecific antibody binding were blocked with 3% hydrogen peroxide in methanol for 25 min followed by 3% normal serum with 3% BSA in PBS (137 mM NaCl, 2.7 mM KCl, 10 mM Na_2_HPO_4_, 2 mM KH_2_PO_4_; pH 7.2–7.4) for 1 h. Goat anti-Pou4f2 (Brn3b), mouse anti-Pou4f1 (Brn3a), rabbit anti-CralBP, goat anti-Sox2, and goat anti-NeuroD1 antibodies (Santa Cruz Biotechnology, Santa Cruz, CA) and rabbit anti-Tbr2 (Chemicon, Temecula, CA) were used at a 1:200 dilution. All antibodies were diluted in 3% normal serum with 3% BSA. Immunoreactivity was visualized using the Vector Laboratories ABC kit (Burlingame, CA) for color visualization, and cells were counterstained with methyl green. To determine cell proliferation levels, 0.1 mg/g of bodyweight of bromodeoxyuridine (BrdU) was administered intraperitoneally into pregnant mice 2 h before they were euthanized by CO_2_ inhalation. BrdU-incorporated cells were detected with a BrdU Staining Kit (Invitrogene, Camarillo, CA).

For immunohistochemical analysis, host mice were euthanized at different times after transplantation. Eyes were dissected and fixed in 10% NBF for 2 h at 4 °C and transferred to 25% sucrose for 16 h at 4 °C before cryosectioning. Twelve-micrometer sections were mounted on Superfrost/Plus microscope slides (Fisher Scientific), which were immunostained with antineurofilament light chain antibody (NFL; Sigma, 1:500), anti-glial fibrillary acidic protein (GFAP; Sigma, 1:1000), anti-Pou4f2 (Santa Cruz Biotechnology,1:400), anti-MAP2 (Sigma, 1:200), anti-neural cell adhesion molecule (NCAM; Sigma, 1:200), anti-Tau (1:200), and antisyntaxin (Sigma, 1:500) for 16 h at 4 °C. For NFL and GFAP staining, slides were chosen when GFP-expressing cells were present, and the slides were processed in the antigen-retrieval solution (Vector Laboratories).

Flatmount double immunohistochemical analysis was performed to detect axons. Five-month-old mice that had undergone transplantation of dissociated RPCs were sacrificed, and eyes were dissected and fixed in 10% NBF for 2 h. The lens was removed from each eye, and the remaining tissue was refixed for 16 h at 4 °C. The eyes were washed with PBS and blocked with 5% normal donkey serum with 0.1% Triton X-100 (Sigma) for 16 h. Anti-NFL antibody (1:250) and anti-Pou4f2 antibody (1:400) were used for 48 h at 4 °C. Eyes were washed four times with PBS containing 0.05% Tween-20 (Sigma) and incubated with Alexa 488- or 594-conjugated donkey anti-goat or anti-mouse secondary antibodies (Invitrogen), respectively, and counterstained with 4',6-diamidino-2-phenylindole (DAPI, Vector Laboratories).

### Alkaline phosphatase staining

Dissociated RPCs from E14.5 embryos of homozygous GFP mice, which had been bred with homozygous *Pou4f2-AP* knock-in mice, were transplanted into one eye of each mouse that had RGC-ablated retinas one month after tamoxifen treatment. Three weeks after transplantation, mice were euthanized, and the eyes were dissected and fixed in 2% paraformaldehyde for 2 h and transferred to 25% sucrose for 16 h at 4 °C before cryosectioning. Twelve-micrometer sections were mounted on Superfrost/Plus microscope slides. A BM Purple alkaline phosphatase (AP) substrate kit (Roche, Indianapolis, IN) was used to visualize AP, and the slides were post fixed in 10% NBF for 2 h. Sections were counterstained with Nuclear Fast Red (Vector Laboratories).

### Photography

Micrographs were obtained with a confocal laser-scanning microscope (Olympus FluoView laser microscope; Olympus Optical Co, Tokyo, Japan) or a digital camera (AxioVision 3.1) mounted on an Axioskop2 microscope (Carl Zeiss, Thornwood, NY). Cranial images were obtained with a Canon Rebel XT digital camera (Canon, Tokyo, Japan). Photographs were assembled in Adobe Photoshop (Adobe Systems, San Jose, CA). Contrast, brightness, and color balance were adjusted to obtain optimal images.

## Results

### Characteristics of donor green fluorescent protein–labeled retinal progenitor cells

We showed previously that *Atoh7*-expressing RPCs make up approximately 30% of the RPC population in E14.5 retinas and that the majority of *Atoh7*-expressing RPCs have exited the cell cycle [19,20]. To follow the fate of the transplanted RPCs, we used retinas isolated from mice carrying a ubiquitously expressed GFP transgene. At E14.5, most cells in the retina are progenitor cells within the progenitor cell layer, although a small number have differentiated into RGCs. As others have shown [10,20], we found that RPCs existed in heterogeneous subpopulations, some of which were proliferating and some post-mitotic. RPCs are exceedingly heterogeneous and continuously change as development proceeds. Each subpopulation expresses transcriptional regulators that specify individual cell types, although there is extensive coexpression among the subpopulations [10,20,21].

**Figure 1:**
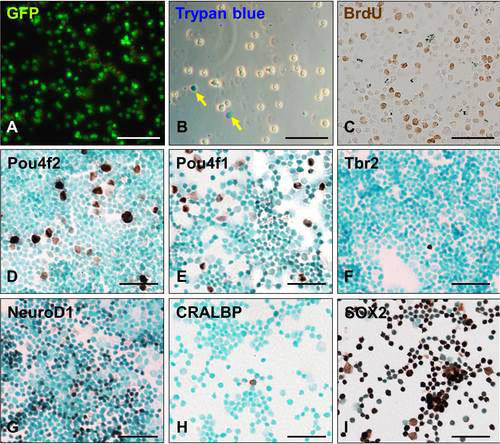
Gene expression in dissociated embryonic green fluorescent protein (GFP)-labeled retinal cells. **A**: GFP expression. **B**: Trypan blue staining. Arrows point to dead cells. **C**-**I**: Immunostaining with antibodies for retinal progenitor cell (RPC) and retinal ganglion cell (RGC) regulatory proteins (brown). The cells were counterstained with methyl green (blue). Scale bars: 100 μm.

Because *Atoh7*-expressing RPCs make up more than 30% of the population of E14.5 cells [19], we used unpurified GFP-expressing RPCs from dissociated retinas (Figure 1A). We injected 10^5^ retinal cells into the vitreous of one eye of Dta mice near the center of the retina. The other eye served as a buffer-injected control. We used mice at two to three months of age, one month after tamoxifen administration. We found that mice older than two to three months were less responsive to the transplanted cells. Eyes, retinas, and optic nerves were dissected two to 16 weeks after the transplantation. More than 95% of the cells in these preparations were viable (Figure 1B). The dissociated retinal cells had signature characteristics of RPCs. Thirty-six percent were actively dividing (Figure 1C), and less than 4% expressed *Pou4f2*, *Pou4f1*, and *Tbr2* (Figure 1D-F). They also did not express *CRALBP*, a glial gene, but 96% expressed *Sox2*, and 29% expressed *NeuroD1* (Figure 1G-I).

**Figure 2:**
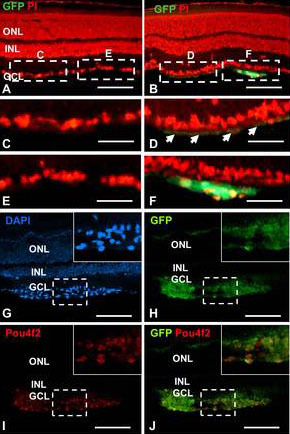
Green fluorescent protein (GFP)-expressing retinal progenitor cells at two to three weeks after intravitreal transplantation. **A**, **B**: Retinal sections from noninjected and injected retinas under a GFP filter with propidium iodide (PI) counterstaining (red). Dotted boxes indicate regions depicted at higher magnification in panels **C**-**F**. **G**-**J**: Retinal sections were immunostained with anti-GFP (green) and anti-Pou4f2 (red) antibodies. Cells in panel **G** were counterstained with DAPI (blue). Dashed boxes indicate regions depicted at higher magnification on each upper-right corner. ONL, outer nuclear layer; INL, inner nuclear layer; GCL, ganglion cell layer. Scale bars: **A**, **B**: 100 μm; **C**-**F**: 50 μm; **G**-**J**: 100 μm.

**Figure 3:**
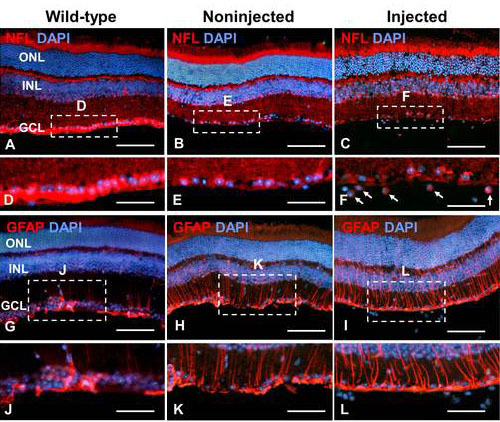
Neurofilament light chain (NFL) and glial fibrillary acidic protein (GFAP) expression in transplanted retinal progenitor cells (RPCs). Retinal sections from wild-type, noninjected, and injected eyes were immunostained with anti-NFL (**A**-**C**) and anti-GFAP (**G**-**I**) antibodies (red). Dashed boxes indicate regions depicted at higher magnification in panels **D**-**F** and **J**-**L**. Sections were counterstained with DAPI. Scale bars: **A**-**C** and **G**-**I**: 100 μm; **D**-**F** and **J**-**L**: 50 μm.

The length of time required for transplanted stem/progenitor cells to differentiate into retina cell types depends on the properties of the transplanted cells and the differentiated cell type. For example, transplanted photoreceptor progenitors differentiated into rods by three weeks [13], while the differentiation of amacrine cells and RGCs can require more time [10]. To determine the optimal time for transplanting E14.5 RPCs, we characterized retinas at two to three, ten, and 16 weeks after injection. At two to three weeks, many transplanted GFP-expressing cells had migrated into the retina immediately below the ganglion cell layer (GCL), but an equal number were in the adjacent interstitial space (Figure 2A-F). Several cells in the GCL coexpressed *Pou4f2* and *GFP*, suggesting that these cells had differentiated into RGCs (Figure 2G-J). We confirmed that GFP-expressing cells were differentiating into RGCs but not Müller glial cells by immunolabeling with NFL (Figure 3A-F and GFAP (Figure 3G-L). However, GFP signals were bleached with the immunostaining procedures that we used for NFL or GFAP antibody staining. To overcome this technical problem, we looked at adjacent sections from Figure 2 and chose cells labeled with GFP to compare them with cells expressing NFL (Figure 3A-F, arrows). We also determined whether the RPCs could differentiate into astrocytes or glial cells; these cells have the potential to dedifferentiate and redifferentiate into nonglial cell types [22]. GFAP expression, a well known indicator for astrocytes and glial cells, was not detected in transplanted RPCs (Figure 3G-L). This result was consistent with the fact that most RPCs at E14.5 had already committed to early cell fates and were not capable of differentiating into later cell types. In addition, gliosis occurs in RGC-ablated retinas [3], but GFAP expression was elevated to the same extent in RPC-injected and buffer-injected retinas, indicating that the transplanted cells were not immunoreactive.

**Figure 4:**
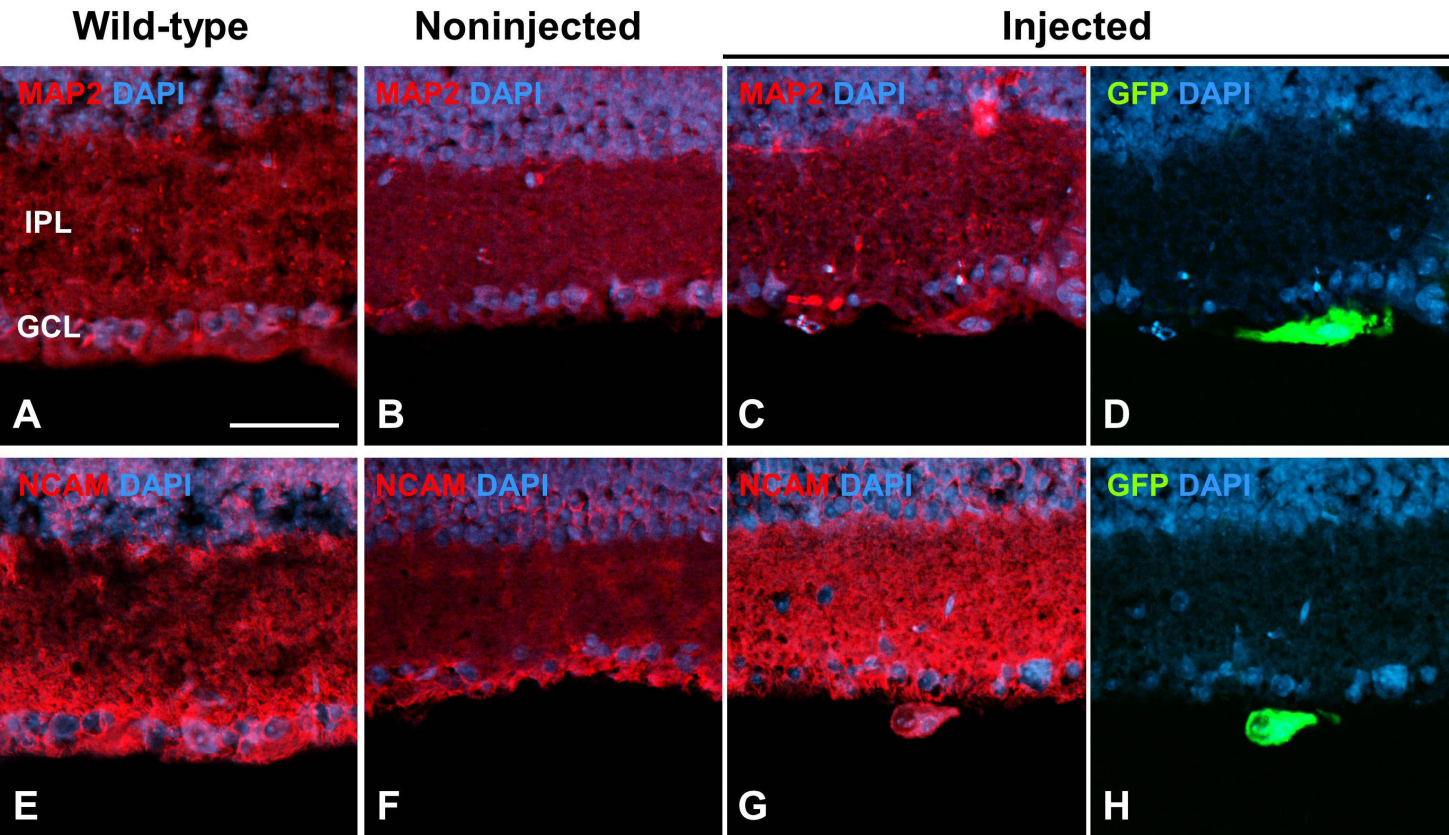
Neuronal differentiation of transplanted retinal progenitor cells (RPCs) inretinal ganglion cell-ablated retinas. Retinal sections two to three weeks after RPC transplantation were immunostained with anti-MAP2 (**A**-**D**) and anti-NCAM (**E**-**H**) antibodies. IPL, inner plexiform layer; GCL, ganglion cell layer. Sections were counterstained with DAPI. Scale bar: 100 μm.

**Figure 5:**
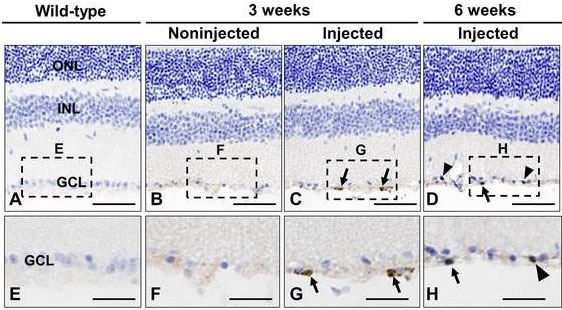
Bromodeoxyuridin (BrdU) pulse-chase-labeled retinal progenitor cells (RPCs) in transplanted RPCs. Retinal sections of wild-type (**A**), noninjected (**B**), and injected eyes (**C**, **D**) were immunostained with anti-BrdU antibody at three or six weeks after transplantation (brown). Dashed boxes indicate regions depicted at higher magnification in panels **E**-**H**: Arrowheads represent BrdU-positive cells that have incorporated into the ganglion cell layer (GCL), and arrows represent BrdU-positive cells that have not incorporated into the GCL (brown). Sections were counterstained with hematoxylin. Scale bars: **A**-**D**: 100 μm; **E**-**H**: 50 μm.

MAP2, Tau, and syntaxin are expressed in dendrites, axons, and ribbon synapses, respectively, during late stages of RGC differentiation [22,23]. A few GFP-labeled cells expressed these genes (Figure 4A-D, Appendix 1), although from the images, it could not be stated with certainty that GFP-positive RGC dendrites and axons were selectively labeled. RGCs derived from transplanted RPCs expressed NCAM as well (Figure 4E-H, Appendix 1). NCAM and its receptor have critical functions in retinal development [24]. GFP signals were undetectable a few weeks after transplantation even though the transplanted cells were still present. We used a BrdU pulse-chase experiment to determine whether cells that once expressed GFP could be incorporated into the retina by examining the number of BrdU-positive cells that entered the depopulated GCL in the transplanted retinas. Three weeks after transplantation, several BrdU-positive cells were detected just underneath the GCL (Figure 5A-C,E-G). By six weeks, however, many more BrdU-labeled cells were in the GCL (Figure 5D,H). Although transplanting RPCs at two to six weeks demonstrated that some RPCs invaded the GCL and differentiated into RGCs, many did not.

**Figure 6:**
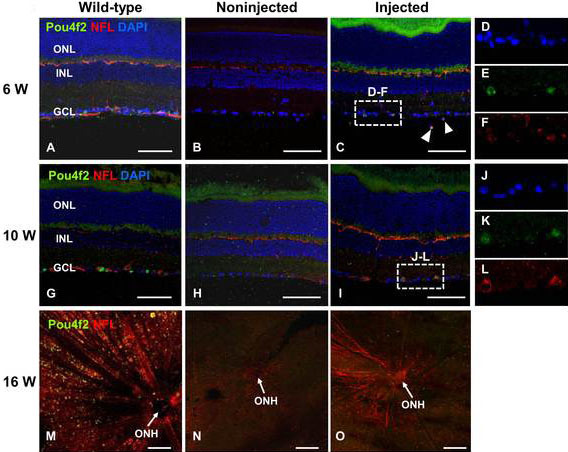
Transplanted retinal progenitor cells differentiate into retinal ganglion cells (RGCs). **A**-**F**: Retinal sections from six (**A**-**C**) and ten weeks (**G**-**I**) after transplantation were immunostained with anti-Pou4f2 (green) and anti-NFL (red) antibodies. Sections were counterstained with DAPI. Dashed boxes indicate regions depicted at higher magnification in panels **D**-**F** and **J**-**L**. Flat-mounted retinas at sixteen weeks after transplantation were immunostained with anti-Pou4f2 (green) and NFL (red) antibodies (**M**-**O**). Arrowheads, Pou4f2-expressing RGCs. ONL, outer nuclear layer; INL, inner nuclear layer; GCL, ganglion cell layer; ONH, optic nerve head. Scale bars: 100 μm.

The time required for transplanted RPCs to differentiate into RGCs appeared to be longer than previously reported by others [10,13]. There were discernible differences between RGC-depleted retinas at six and ten weeks after transplantation, with many more *Pou4f2*- and NFL-expressing cells in the GCL at ten weeks (Figure 6A-L). In particular, the processes extending from *Pou4f2*-NFL coexpressing cells expressed NFL (Figure 6J-L). By 16 weeks, NFL-labeled axons were detected bundling together in a radial pattern entering the optic nerve head (Figure 6M-O). These results implied that the axons were projecting considerable distances beyond the optic nerve head into the damaged nerve.

### Optic nerve regeneration in retinal ganglion cell–depleted mice

**Figure 7:**
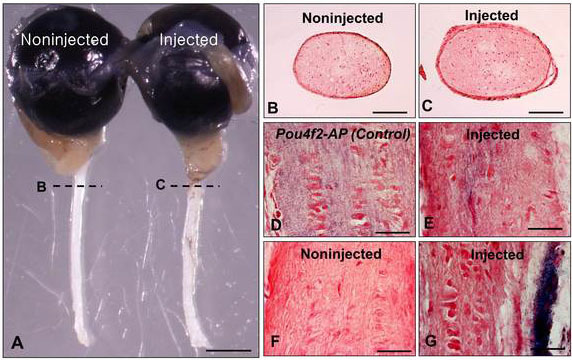
Alkaline phosphatase (AP) expression in retinal ganglion cell axons three weeks after retinal progenitor cell transplantation. **A**: Dissected eyes and optic nerves. **B**, **C:** Cross-sections of optic nerves from noninjected and injected eyes. **D**-**G**. AP stained Pou4f2-AP (**D**) buffer-injected (**F**) and injected (**E**, **G**) optic nerves. Sections were counterstained with Nuclear Fast Red. Scale bars: **A**: 1 mm; **B**, **C**: 100 μm; **D**-**G**: 25 μm.

In contrast to other retinal neurons, RGC axons form thick fibers that extend long distances. We injected dissociated RPCs into the vitreous of one eye of RGC-ablated mice and determined the extent of RGC axon formation in optic nerves up to 16 weeks after RPC transplantation. Three weeks after tamoxifen administration, the optic nerve had not completely degenerated. However, the optic nerves with transplanted RPCs were noticeably thicker than those without transplanted RPCs, implying that some axon regeneration was occurring (Figure 7A-C).

Although many GFP-labeled RPCs differentiated into RGCs, the overall efficiency of transplantation (number of RPCs injected/number of RPCs differentiating into RGCs) was only about 10%. It was therefore possible that the axons within the optic nerve did not originate from the transplanted cells. Transplanted neuronal progenitors can produce neuroprotective factors that suppress cell death [25]. Approximately 10% of RGCs in the adult retina do not express *Pou4f2* [26], and Dta-mediated cell death would not occur in these cells. The protective environment imparted by the RPCs might enhance the survival of residual endogenous RGCs and promote the extension of their axons into the optic nerve.

To determine whether the optic nerve axons were coming from the transplanted RPCs, we used a *Pou4f2* allele in which the gene for AP was inserted into the *Pou4f2* locus [27]. If the transplanted RPCs were differentiating into RGCs, then axons of differentiated cells would express AP. We bred *Pou4f2-AP* knock-in mice with GFP mice and injected one eye of the offspring with GFP-labeled RPCs and the other with buffer. Longitudinal sections of axons projecting from eyes transplanted with RPCs expressed AP, although the AP-labeled axons were disorganized and considerably fewer in number than those of the wild-type controls (Figure 7D,E). Buffer-injected eyes expressed only background levels of AP in their optic nerves (Figure 7F). Sections in other places also showed AP labeling (Figure 7G). In these places, AP-expressing axons were mainly distributed along the outer edges of the optic nerve, although weak labeling was seen internally as well (Figure 7G). The optic nerve from *Pou4f2-AP* knock-in control mice was uniformly labeled, while no labeling was detected in buffer-injected eyes (Figure 7D,F). These results demonstrated that at least some of the transplanted RPCs were directly responsible for generating RGC axons. The results also indicated that the apparent axon regeneration was not uniform across the optic nerve.

**Figure 8:**
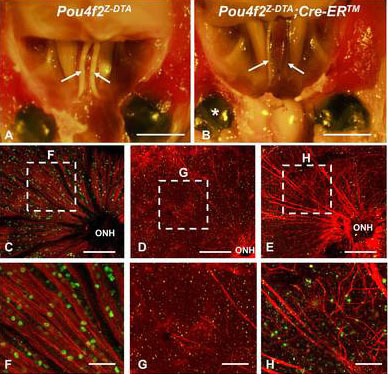
Retinal ganglion cell axon restoration and optic nerve regeneration in retinal progenitor cell (RPC)-transplanted eyes. **A**, **B**: Cranial images from tamoxifen-treated control- (**A**) and RPC-injected (**B**) mice sixteen weeks after transplantation. **C**-**E**: Flat-mounted retinas from control (**C**) noninjected (**D**) and injected (**E**) eyes with anti-Pou4f2 (green) and anti-NFL (red) antibodies. Dashed boxes depict regions at higher magnification in panels **F**-**H**: Arrows, optic nerve; asterisk, RPC-transplanted eye, OHN, optic nerve head. Scale bars: **A**, **B**: 3 mm; **C**-**E**: 200 μm, **F**-**H**: 50 μm.

By four months after tamoxifen injection, the optic nerves of the Dta mice had degenerated, with only a few abnormal fibers surrounded by the extracellular sheath (Figure 8A,B). The optic nerve of the eyes transplanted with RPCs showed statistically significant increases in optic nerve thickness and axon bundles 16 weeks after injection in six of eleven mice. In optic nerves from mice injected with donor cells, two of 11 mice were about threefold thicker than the uninjected eyes (injected, 0.48±0.01 mm^2^; noninjected, 0.16±0.03 mm^2^) but not as thick as the wild-type optic nerves (0.72±0.10 mm^2^; Figure 8A,B). In four of 11 mice, the area of the optic nerve from the injected eye was 1.38 times thicker (injected, 0.25±0.03 mm^2^; noninjected, 0.18±0.02 mm^2^; p=0.014). However, in five of 11 mice, there was no significant difference (injected: 0.16±0.02 mm^2^; noninjected: 0.17±0.01 mm^2^; p=0.52). The overall p value for the 11 mice was 0.055, suggesting a strong trend toward increased optic nerve thickness after RPC injection. The reason for the variation was unclear, although it was likely caused by subtle differences in the depth and point of contact of the injection. Possible sources contributing to the variation could be differences in retinal damage from one injection to another or the effectiveness of the injected cells to distribute beyond the injection point.

The flatmount retinas showed extensive axon bundling within many axons projecting into the optic nerve head (Figure 8F-H). Although many axons were fasciculated and well directed, others had abnormal branching and were disorganized (Figure 8C-H). We also noted that the majority of RGCs and axons derived from the transplanted RPCs originated from the site of injection near the center of the retina (Figure 8E). This suggested that most RPCs entering the GCL were not able to migrate from their original point of entry.

**Figure 9:**
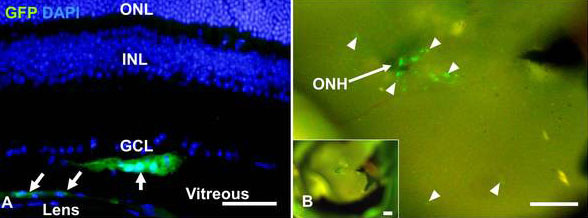
Green fluorescent protein (GFP)-expression in transplanted cells after intravitreal injection. Retinal section (**A**) and inside image (**B**) at two to threeweeks after transplantation. Slides were counterstained with DAPI (blue in panel **A**). Arrows and arrowheads indicate transplanted cells (green). ONL, outer nuclear layer; INL, inner nuclear layer; GCL, ganglion cell layer; ONH, optic nerve head. Scale bars: 100 μm.

**Figure 10:**
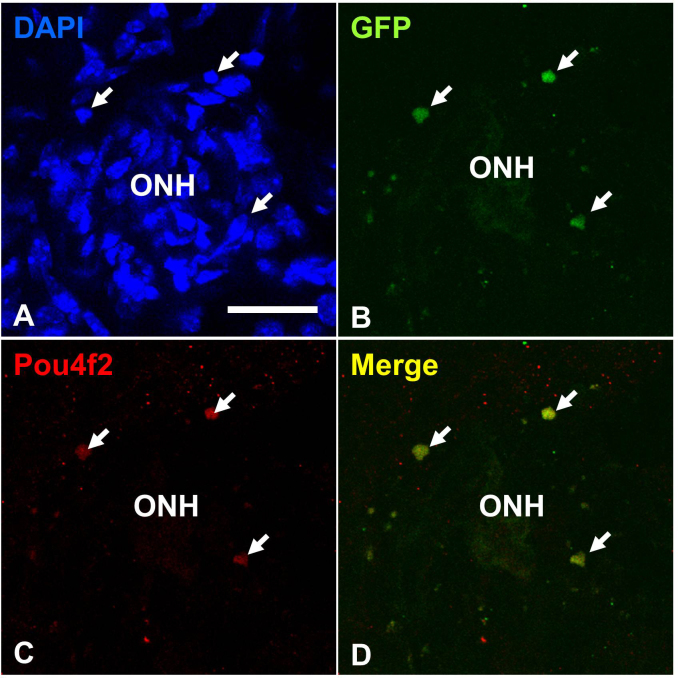
Green fluorescent protein (GFP)- and Pou4f2-expression in transplanted cells six weeks after intravitreal injection. Retinal flat mounts with DAPI (**A**), anti-GFP antibody (**B**) anti-Pou4f2 antibody (**C)**: and merged imaged with panel **B** and **C** (**D**). Arrows indicate transplanted cells. ONH, optic nerve head. Scale bars: 50 μm.

Similar results were found when we analyzed the dissected eyes and sectioned retinas for GFP expression. Usually, two to three weeks after transplantation, most cells were detected more in the intravitreal area close to the optic nerve head (Figure 9A,B). We confirmed that many GFP-expressing transplanted cells also expressed Pou4f2 (Figure 10).

## Discussion

### Model hosts and stem/progenitor cell donors for optic nerve repair

We used a genetic ablation mouse model as the host and an enriched embryonic progenitor cell population as the donor to achieve partial restoration of RGCs and regeneration of optic nerves. The Dta mice were obviously not suitable for experiments aimed at enhancing RGC survival. However, our goal was to use the Dta mice as a new tool for RPC or other stem cell replacement experiments. We found that *Atoh7*-expressing RPCs were capable of populating the GCL, expressing RGC genes, differentiating, and contributing to increased axon bundling and optic nerve thickness. However, the number of GFP-labeled RPCs contributing to RGC differentiation made it likely that the injected RPCs were acting autonomously and non-autonomously. In fact, it was likely that the improvement in the thickness of the degenerated optic nerves represented the ameliorative effects of the transplanted cells. If true, it will be important to determine the underlying basis of the effect and whether embryonic RPCs as opposed to other neuronal-derived cells have unique characteristics in adult retinas.

Transplanted cells that have been used to repair damaged retinas include intact sheets of embryonic retinas, dissociated retinal cells, multipotent RPCs, neural progenitors derived from the hippocampal progenitor zone, human embryonic stem cells, and cells derived from the pigmented ciliary epithelium [10]. In general, the most successful of these cell transplantation experiments restore photoreceptor cells, not RGCs, amacrine, horizontal, or bipolar cells [28]. Although several reports have described some success in using stem cells or progenitor cells to restore RGCs and repair degenerate optic nerves, most have focused on the novelty of the cells being used for the transplantation experiments rather than the effectiveness of the transplantation to regenerate damaged optic nerves [1,2]. Although many reports have demonstrated that transplanted cells can differentiate into RGCs, few of these studies have addressed optic nerve restoration per se [2,10]. In addition, the low efficiency of transplanted cells differentiating into fully formed RGCs has been and remains a chronic problem.

As with a previous study of rod photoreceptor cells [13], our study of RGCs demonstrated the importance of choosing a time during embryogenesis when the progenitor pool, in this case *Atoh7*-expressing RPCs, is maximal.

### The environment of the embryonic and adult retina

In many adult tissues, the identity and properties of stem cell niches are well known [29-31]. Most relevant to our study, stem cell niches have been identified in the brain and other parts of the nervous system [31,32]. These niches offer welcoming environments for transplanted cells, viruses, and bioengineered tissues. In the adult retina, what appear to be quiescent stem cells and their corresponding niche in the periphery of the retina have been identified by continuous repassaging of neurosphere cultures [33]. However, the mechanism keeping these putative stem cells in their quiescent state is still unclear, and ways to reactive them in vivo have yet to be developed [2]. The ability of Müller glial cells to dedifferentiate into stem cells offers a possible solution to the adult stem cell problem, but the developmental potential for Müller glial stem cells to differentiate into retinal cell types other than photoreceptor cells is limited [21].

During embryonic and postnatal retinal development, progenitor cells are continuously generating retinal cell types. The intrinsic program of RPCs and the extrinsic environment of the developing retina are dynamic in space and time [4,5]. Successful RPC transplantation approaches require not only an enriched population of progenitors but also a nurturing environment in the adult retina in which to differentiate. In adult mice, the inner limiting membrane represents a significant barrier for incorporating intravitreally injected donor cells [34,35]. However, mechanically removing this membrane or puncturing it with the injection needle can provide donor cells with a better environment after transplantation [36]. To support this view, when we did not puncture the inner limiting membrane, the injected cells remained in a large clot of cells in the vitreous body of the retina (data not shown).

Moreover, neuronal progenitor cells and other stem cells are known to secrete a wide variety of neuroprotective factors [24]. The transplanted RPCs in our experiments may be contributing to enhancing their own survival as well as residual non-*Pou4f2*-expressing RGCs in an otherwise hostile environment for foreign cells. To further enhance this self-survival mechanism, coinjection of RPCs with neuroprotective factors might contribute to improved efficacy [37]. To date, incubating GFP-labeled RPCs with ciliary neurotrophic factor (CNTF; 5 ng/ml), brain-derived neurotrophic factor (BDNF; 10 ng/ml), and forskolin (5 μg/ml) have not improved the efficiency of transplantation (data not shown). Other neuroprotective approaches include coculturing progenitor cells with other cells known to promote survival or suppress the immune response [34].

### Improving the efficacy of retinal progenitor cell transplantation

Our experiments did not result in efficient restoration of the optic nerve. Although many axons derived from transplanted RPCs bundled together and extended relatively long distances, most were abnormal. Electroretinograms of transplanted eyes failed to produce signals above those in nontransplanted eyes, indicating that the thickened optic nerves were not capable of transmitting electrical signals [38]. The transplanted RPCs did not distribute throughout the GCL but remained near the injection site in the central retina. This suggested that migration of proliferating RPCs was impeded. In our experiments, we injected RPCs into a single site because the trauma induced by injections into multiple sites damaged the tissue. In the developing retina, RPCs migrate into the emerging GCL by traveling along thin processes in apical and basal directions [39]. This complex process cannot be reproduced in adult retinas, but developing methods to enhance the dispersal of injected RPCs could improve the efficacy of cell replacement for inner retinal neurons.

Optic nerve degeneration commonly occurs in older individuals. Our initial attempts to restore RGCs were performed with older mice that had complete optic nerve degeneration in the skull base at the time of transplantation. In these cases, transplanted cells were found in the GCL, but they did not contribute to optic nerve regeneration. Only when we transplanted RPCs into younger mice and waited 16 weeks before euthanizing the mice for analysis did we find significant optic nerve regeneration. Our approach emphasizes the importance of time for obtaining meaningful information with older mice. It is possible that reactive gliosis in severely damaged retinas progressed to a point that it prevented the differentiation of the donor cells into RGCs. Moreover, complete optic nerve degeneration at the time of injection may prevent the transmission of neurotrophic factors from the brain to the retina. Retrograde labeling of RGC-ablated retina indicated that as the optic nerve degenerated over three months, there was a large reduction in DiI labeling in the retina [3]. Thus, determining the right injection time is a critical prerequisite in any transplantation procedures involving optic nerve regeneration.

We injected E14.5 RPCs because this is when RGCs are beginning to differentiate. RPCs from P0 or P1 retinas were not as effective as those from E14.5. However, we have yet to inject RPCs from earlier stages, which might improve the efficacy. In addition, we have recently been able to purify *Atoh7*-expressing RPCs at E13.5 using fluorescence activated cell sorting (FACS, Beckman Coulter, Brea, CA). These purified cells might also improve the efficiency of injected donor cells into host retinas. Indeed, Sun et al. [40] found that E13.5 progenitor cells had a greater ability to differentiate into RGCs in culture than E17.5 progenitor cells.

Recent approaches for inducing stem cells to grow into a variety of adult and embryonic cells use viral cocktails of genes encoding stem cell-expressing transcription factors to promote stem cell proliferation [41]. The gene regulatory network operating in RGC development requires several transcription factors besides *Atoh7* for differentiation to occur. Although *Pou4f2* and *Isl1* were activated in transplanted RPCs, introducing them into transplanted cells beforehand and coupling their expression to a conditionally inducible promoter could lead to more productive RGC differentiation.

## Appendix 1. Neuronal differentiation of transplanted RPCs in RGC-ablated retinas.

To access the data, click or select the words “Appendix 1.” This will initiate the download of an image (jpg) archive that contains the file. Retinal sections 2-3 weeks after RPC transplantation were immunostained with anti-MAP2 (A–C), anti-NCAM (D–F), anti-tau (G–I), and anti-syntaxin (J–L) antibodies. GCL, ganglion cell layer. Scale bar: 100 μm.
